# Population attributable fraction of type 2 diabetes due to physical inactivity in adults: a systematic review

**DOI:** 10.1186/1471-2458-14-469

**Published:** 2014-05-18

**Authors:** Hashel Al Tunaiji, Jennifer C Davis, Dawn C Mackey, Karim M Khan

**Affiliations:** 1 Centre for Hip Health and Mobility, University of British Columbia, Vancouver Coastal Health Research Institute (VCHRI), British Columbia, Canada; 2 Zayed Military Hospital, Abu Dhabi, United Arab Emirates; 3 Department of Biomedical Physiology and Kinesiology, Simon Fraser University, British Columbia, Canada; 4 Centre for Clinical Epidemiology and Evaluation, School of Population and Public Health, University of British Columbia, British Columbia, Canada; 5 Aspetar - Orthopaedic and Sports Medicine Hospital, Doha, Qatar; 6 Department of Family Practice, Faculty of Medicine, University of British Columbia, British Columbia, Canada; 7 Centre for Hip Health and Mobility, Robert H.N. Ho Research Centre, 769-2635 Laurel Street, Vancouver, BC V6H 2K2, Canada

**Keywords:** Population attributable fraction (PAF), Physical inactivity, Type 2 diabetes (DM-2), Systematic review

## Abstract

**Background:**

Physical inactivity is a global pandemic. The population attributable fraction (PAF) of type 2 diabetes mellitus (T2DM) associated with physical inactivity ranges from 3% to 40%. The purpose of this systematic review was to determine the best estimate of PAF for T2DM attributable to physical inactivity and absence of sport participation or exercise for men and women.

**Methods:**

We conducted a systematic review that included a comprehensive search of MEDLINE, EMBASE, SportDiscus, and CINAHL (1946 to April 30 2013) limited by the terms adults and English. Two reviewers screened studies, extracted PAF related data and assessed the quality of the selected studies. We reconstructed 95% CIs for studies missing these data using a substitution method.

**Results:**

Of the eight studies reporting PAF in T2DM, two studies included prospective cohort studies (3 total) and six were reviews. There were distinct variations in quality of defining and measuring physical inactivity, T2DM and adjusting for confounders. In the US, PAFs for absence of playing sport ranged from 13% (95% CI: 3, 22) in men and 29% (95% CI: 17, 41) in women. In Finland, PAFs for absence of exercise ranged from 3% (95% CI: -11, 16) in men to 7% (95% CI: -9, 20) in women.

**Conclusions:**

The PAF of physical inactivity due to T2DM is substantial. Physical inactivity is a modifiable risk factor for T2DM. The contribution of physical inactivity to T2DM differs by sex; PAF also differs if physical inactivity is defined as the absence of ‘sport’ or absence of ‘exercise’.

## Background

Physical inactivity, a global pandemic [1], is one of the most serious public health problems of the 21^st^ century in terms of consequences and cost [2-4]. Annually, the global mortality attributable to physical inactivity is approximately 3.3 million [5]. Globally, physical inactivity is identified as the fourth leading risk factor for mortality among adults [5]; it is an independent risk factor for major chronic diseases [6,7]. Physical inactivity is also associated with substantial economic burden across the globe, accounting for instance annual direct cost of SFr 1.6 billion in Switzerland (1999 prices) to $US 24 billion in the USA (1999 prices) [4].

Type 2 diabetes mellitus (T2DM) also imposes a significant health and economic burden on North American health care system [8]. In the US alone (2011), the age–adjusted incidence increased 117% from 3.5 to 8.3 per 1,000 persons between 1980 and 2011 [9]. The cases of T2DM were projected to increase from 12 million in 2000 to 39 million by 2050 (i.e. a prevalence increase from 4.4% to 9.7% in 2050) [10]. The direct costs associated with T2DM was approximately $US 44.1 billion per year or almost $US 6000 per person per year (1997 prices) [11]. Furthermore, the cost of T2DM attributable to physical inactivity (absence of leisure-time activity) ranged from $US 1.90 billion to $US 13.20 billion per year (2007 prices) [12].

Physical activity benefits at least 23 different health conditions [13,14]. Despite this, fewer than 50% of the people engage in sufficient physical activity to reap such benefits [15,16]. Prospective studies demonstrate that physical inactivity is an independent and modifiable risk factor for T2DM [17]. Specifically, physical activity interventions reduced the risk of developing diabetes [18-20].

A method of quantifying the burden of T2DM attributable to physical inactivity is population attributable fraction (PAF). PAF takes into account the degree of association between a risk factor and the incidence of a disease (i.e., relative risk) and the public health importance of this risk factor at a population level. Specifically, PAF estimates the proportion of disease cases (i.e., T2DM cases) that are attributable to a risk factor of interest (i.e., physical inactivity) among all disease cases in a population [21].

To date, PAF estimates for the excess cases of T2DM vary from 3% due to lack of exercise in Finland to 40% in Canada due to lack of moderate-vigorous physical activity [13-15]. Some of this variability is due to variation in calculating PAF based on age-, gender-, region-specific factors. But there has been no systematic review that has assessed the PAF of T2DM attributable to physical inactivity in men and women. Also, none has used recent advances in PAF as outlined by Laaksonen [13]. Examining the quality of these discrepant estimates and underlying reasons for the observed variation is important as it will provide policymakers with a guide to which of the original studies should carry most weight. Hence, our primary objective was to quantify the PAF of T2DM attributable to physical inactivity and absence of sport participation or exercise for men and women.

## Methods

### Data sources and search strategy

**Figure 1:**
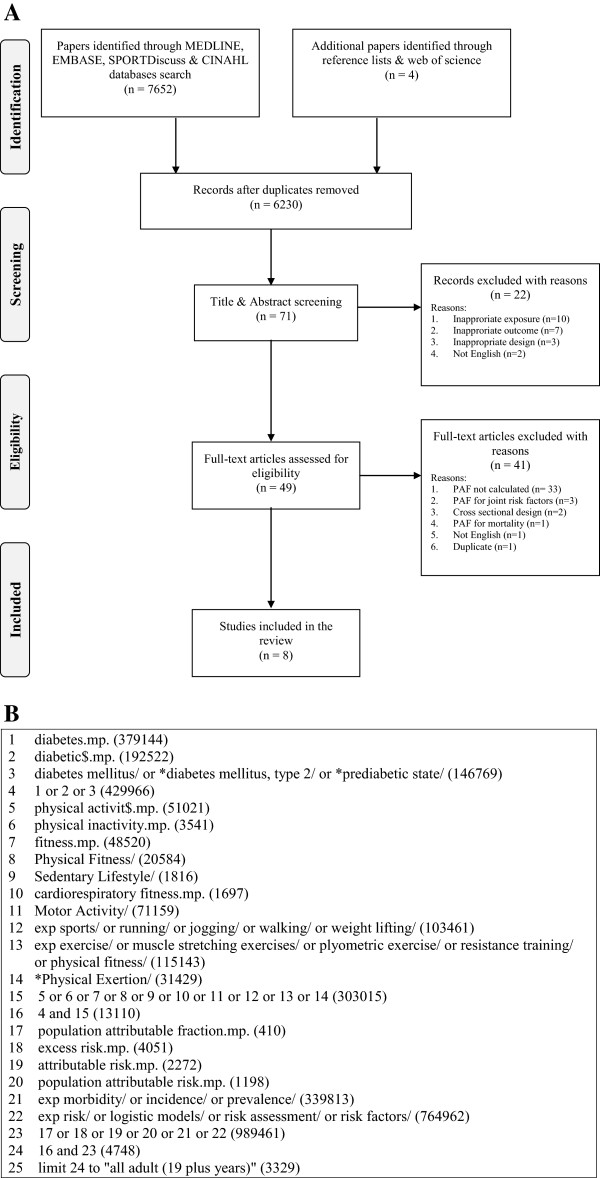
A Flow diagram showing study selection (1A) and database search (1B) for systematic review of studies on population attributable fraction (PAF) of type 2 diabetes (T2DM) due to physical inactivity in adults.

In accordance with the Preferred Reporting Items for Systematic Reviews and Meta-Analysis (PRISMA) statement [22] and Cochrane Collaboration guidelines [23], we [HAT, JCD, KMK] conducted a comprehensive search of MEDLINE, EMBASE, SportDiscus, and CINAHL. We limited our search results to adults aged 19-65 years and studies published in English. We included search terms the MeSH headings: diabetes, physical activity, fitness, risk assessment. The search strategy detailed in Figure 1(B) includes studies published between 1946 and April 30 2013. We manually searched all references of articles selected for full text review to identify additional relevant papers.

### Study selection and eligibility criteria

We (HAT, JCD) included peer reviewed, published studies that (i) estimated PAF or population attributable ratio (PAR) using modeling on raw data from a prospective cohort design or (ii) published adjusted relative risk (RRadj) and prevalence of the risk factor of interest – physical inactivity [24]. Of note, review studies were included if their RRadj estimates were based on prospective cohort data. Based on title and abstract review, we excluded studies that: 1) used an exposure unrelated to physical inactivity), 2) used an outcome that was not T2DM, 3) used an inappropriate study design for estimating PAF/PAR (i.e., cross-sectional, case-control or retrospective studies). Based on full text review, we excluded studies that: 1) did not contain a PAF estimate, 2) did not detail the independent contribution of physical inactivity, 3) used an inappropriate study design for estimating PAF/PAR (i.e., cross-sectional, case-control or retrospective studies), 4) the primary outcome was not T2DM, 5) were duplicates. Eight full-text articles met the inclusion criteria – four from our search strategy and four from our review of the reference lists of all articles selected for full text review. All discrepancies were resolved by discussion and consultation with a co-author (KMK). Figure 1(A) details the process of study selection for this systematic review.

### Data extraction

Two raters (HAT, JCD) independently extracted data from each study and any discrepancies were discussed and reviewed by a third party (KMK). We developed a list of data extraction topics for the studies included in this systematic review (Additional file 1: Table S1 and Additional file 2: Table S2). These items were: author’s name, year of publication, country, journal name, study design, sample size, sample characteristic, length of follow up, operational definition for exposure (physical inactivity), operational definition for outcome (T2DM), level of adjustment for confounders, PAF estimates and calculation method used to estimate PAF (Additional file 1: Table S1 and Additional file 2: Table S2).

### Exposure, outcome and outcome measures for data synthesis

Our primary exposure of interest for population attributable fraction (PAF) estimates was physical inactivity. Physical inactivity was defined as total physical activity insufficient to meet recommended guidelines, that is ≤ 150 minutes of moderate-intensity or ≤ 75 minutes of vigorous-intensity aerobic physical activity per week in bouts of at least 10 minutes duration accumulated across occupational, transport-related, domestic or leisure-time domains [5]. Leisure –time activity domains includes exercise, sport and unstructured recreation [14,25,26]. Exercise [27] is a planned, structured and repetitive physical activity with the purpose of improving and/or maintaining physical fitness. i.e. both exercise and sport are subsets of leisure-time domain and are not interchangeable [27]. Sport is a subset of exercise undertaken either individually or as a part of a team where participants adhere to a common set of rules or expectation and a defined goal to win [28]. Physical inactivity was either self-reported or directly measured by accelerometry.

Our primary outcome of interest for estimating population attributable fraction (PAF) was T2DM defined as: 1) hyperglycemia ascertained by fasting plasma glucose ≥ 7.0 mmol/l (126 mg/dl) or 2-h plasma glucose ≥ 11.1 mmol/l (200 mg/dl) or 2) self report with validation from a registry, medical record or reimbursement plan [29,30].

Population attributable fraction (PAF) or proportion (PAR) was defined as the excess number of cases of T2DM attributable to physical inactivity or low physical fitness that is estimated by the following formula or one of its variant [31]:

$$\begin{array}{l} \mathrm{PAF}\;\frac{P_{e}\left(\mathrm{RRadj}‒1\right)}{\left(\mathrm{RRadj}‒1\right)}\times \;100\\ \; \end{array}$$

Where, P_e_ is population prevalence of exposure and RRadj is an adjusted relative risk.

Of note, we estimated the confidence intervals (95% CI) for PAF using the substitution method when these data were not reported [32]. All calculations done by the authors are labeled with an ‘^a^’ in Additional file 2: Table S2. This method used the upper and lower limits of RR in attributable risk (AR) formula.

Due to study design, sample and analytic heterogeneity, a meta-analysis of these data to determine PAF for T2DM was not conducted.

### Quality assessment

Because our systematic review consisted of both prospective cohort studies and reviews, a published quality assessment checklist suitable for this study was not available. Therefore, we developed a seven-item quality assessment form. This form was created after reviewing potentially relevant checklists such as the STROBE [33,34]. From these examples, we created and modified questions relevant to assessing the quality of the PAF estimates included in this systematic review. The questions were structured so that they could be applied across all included studies and study designs (Additional file 3: Table S3). All quality assessment questions were reviewed by an expert in the field. This quality assessment was not validated. We used dichotomized answers (+: yes, -: no) for the quality assessment questions to create a score out of 7. Two authors (JCD, HAT) independently evaluated each study and any discrepancies were discussed and reviewed by a third author (KMK). Below, we outline each of the criteria included in the quality assessment.

#### Quality assessment questions

##### Question 1: Was a clear definition provided for the exposure (physical inactivity)?

Physical inactivity was defined as the total activity that does not meet the recommended guidelines of ≤ 150 minutes of moderate-intensity or ≤ 75 minutes of vigorous-intensity aerobic physical activity per week in bouts of at least 10 minutes duration accumulated across occupational, transport-related, domestic or leisure-time activity domains [5].

##### Question 2: Was the exposure (physical inactivity) measured objectively?

Physical inactivity can either be measured subjectively using validated self reported questionnaires or objectively using accelerometers [35].

##### Question 3: Was a clear clinical definition provided for the outcome (type 2 diabetes)?

T2DM was defined using hyperglycemia cutoffs as listed above.

##### Question 4: Was the outcome ascertained by objective measures or if self reported confirmed by other measures?

The current diagnostic criteria is requires a fasting plasma glucose ≥ 7.0 mmol/l (126 mg/dl) or a 2-h plasma glucose ≥ 11.1 mmol/l (200 mg/dl) [29,30].

##### Question 5: Was the analysis based on raw data from a prospective cohort study?

One of the PAF assumptions is causality; therefore, only prospective studies were deemed appropriate for estimating PAF [21].

##### Question 6: Was the follow up time provided?

PAF is subject to follow up time bias [21]. Specifically, a shorter follow up time is associated with an overestimated PAF while a longer follow up time is associated with an underestimated PAF.

##### Question 7: Was population attributable fraction (PAF) or proportion (PAR) fully adjusted?

PAFs are subject to confounding bias [24]. The partial adjustment method is a popular method of calculating PAF. It uses published adjusted RR and prevalence of exposure in this formula [24]:

$$\mathrm{PAF}=\frac{P_{e}\left(\mathrm{RRadj}‒1\right)}{\mathrm{RRadj}‒1}\times \;100$$

The partially adjusted method can yield severely biased PAF estimates [36] because the confounding variables are not adequately adjusted. For instance, incomplete adjustment for confounding by age and sex can lead to 17% overestimation in PAF [36]. Therefore modeling all known confounders (i.e., full adjustment modeling method) is a better approach [24].

## Results

### Overview of studies

After critical review of the 49 full text manuscripts, eight studies met our inclusion criteria (Figure 1A, Additional file 1: Table S1 and Additional file 2: Table S2). There were distinct variations in quality across studies with respect to defining and measuring physical inactivity, defining and measuring T2DMand adjusting for confounders in the final model for calculating PAF and follow up time (Additional file 1: Table S1 and Additional file 2: Table S2). Of the eight studies, three focused on the exposure of ‘total physical inactivity’ [26,37,38], three on leisure-time activity and two on subsets of leisure-time activity - specifically ‘exercise’ [39] and ‘sport’ [40] (Additional file 2: Table S2). Of the eight studies, two described three different prospective cohorts and six were reviews of published data. The two prospective cohort studies (included three prospective cohorts) [14] estimated PAF using full adjustment modeling. The six reviews estimated PAF using published data of adjusted relative risk (RRadj) from previously published cohort studies and estimated the prevalence of physical inactivity (Pe) from cross-sectional data. Physical inactivity was self- reported in all studies except one that used data on prevalence of physical inactivity measured by accelerometry [37].

### Prospective cohort studies (2 studies, 3 prospective cohorts)

The three prospective cohorts scored the highest on quality assessment, Additional file 3: Table S3. The PAF for physical inactivity ranged from 3% (95% CI: -11, 16) to 29% (95% CI: 17, 41). In Finland, the PAF from two prospective cohort studies for exercise, a subset of leisure-time domain, ranged from 3% (95% CI: -11, 16) to 7% (95% CI: -9, 20) [13]. The cumulative incidence ranged from 2.6 to 3.9 per 100 people, the adjusted relative risk (RRadj) ranged from 1.28 (95% CI: 0.99, 1.48) to 1.35 (95% CI: 0.97, 1.6) and the prevalence of physical inactivity (Pe) ranged from 24.1% and 36.5%. In the USA, the PAF for sport, subset of leisure-time domain, to range from 13% to 29%: 13% (95% CI: 3, 22) in men and 29% (95% CI: 17, 41) in women [40]. The cumulative incidence was 7.6 per 100 person, the adjusted relative risk (RRadj) was 1.21 (95% CI: 1.1, 1.35) for men and 1.43 (95% CI: 1.21, 1.68) for women and the prevalence of physical activity (Pe) was 55.2% for men and 66.3% for women.

### Country-specific reviews on published data (4 studies)

The PAF estimates from these four studies ranged from 20.1% (17.8, 30.1) [37] to 39% (95% CI: 35.9, 41.7) [38] for total physical inactivity and 19.9% (95% CI: 11, 27.1) [41] to 21.1% (16.5, 25.2) [42] for leisure-time activity. The 95% confidence intervals were constructed for all PAF estimates using the substitution method [32]. The adjusted relative risk (RRadj) ranged from 1.24 (95% CI: 1.1, 1.39) to 1.74 (95% CI: 1.65, 1.83) and the prevalence of physical inactivity ranged from 19.8% to 82% for men and 26.8% to 86.3% for women. The ranges of PAF, RRadj and Pe estimates from these country-specific studies were narrower than estimates generated from the three prospective cohort studies.

### Global review on published data (2 studies)

In general, the global review studies [14,26] reported lower PAFs than the country-specific reviews and the prospective cohort studies except for Finland. The review studies had different definitions for physical inactivity [14,26]. Bull [26] defined physical inactivity as total physical inactivity while Lee [14] referred to leisure-time activity alone. Further, these two reviews used different formulas containing different denominators to calculate PAF from previously published data (Additional file 1: Table S1 and Additional file 2: Table S2) [14,26]. Bull’s [26] PAF estimates for total physical inactivity ranged from 5.2% (95% CI: 2.2, 8.2) in Canada to 13% (95% CI: 4.8, 16.6) in Finland for total physical inactivity while Lee [14] estimated PAFs for leisure-time to range from 7% (95% CI: 0.8, 14.4) in Canada to 10.7% (95% CI: 5.4, 16.8) in South Africa. In one review [26] the 95% CI intervals were not reported therefore we reconstructed them using the substitution method [32]. The adjusted relative risk (RRadj) 1.24 (1.1, 1.39) and the prevalence of physical inactivity ranged from 23% to 61%.

## Discussion

### A review of the variation that exists in PAF across the existent literature

The PAF estimates for T2DM that is attributable to physical inactivity varied widely from 3%-39% across studies (Janssen & Laksoonen). As determined from the performance on our quality assessment, the best quality data in this systematic review suggest that the PAF of T2DM due to physical inactivity in the USA for a non sport participant (never engaged in strenuous sports) ranged from 13% (95% CI: 3, 22) in men and 29% (95% CI: 17, 41) in women. In Finland, Finland, the PAF of T2DM due to physical inactivity for the occasional exerciser (≤30 min/day, subset of leisure-time activity domain) ranged from 3% (95% CI: -11, 16) to 7% (95% CI: -9, 20). The PAF estimates for T2DM attributable to physical inactivity varied widely. Specifically, further variation is notable across study design, countries and sex. Such divergence may be explained by the distinct inconsistency in quality across studies. Below we elaborate on how two categories relating to study methodology and statistical analysis contribute to the observed variation in PAF estimates.

### Analysis of the potential explanations for the demonstrated variation in PAF

Two main factors explain the wide variation we observe in the PAF estimates for T2DM attributable to physical inactivity: heterogeneous study methodology (i.e., study design, exposure and outcome measurement) and statistical methodology.

### Methodology

#### Choice of study design

The choice of study design is a key factor that may explain substantial variation PAF estimate. More recently, methodological advances demonstrate that prospective cohort studies are preferable for PAF estimation because the calculations rely on censored time to event data [43,44,28]. Historically, there is a large body of literature estimating PAF from case-control and cross sectional data [24]. For example, only two of the eight studies included in this systematic review reported three prospective cohort studies that were designed to estimate PAF as a primary outcome measure. As such, we observed wide variation in PAF estimates due to fundamental differences in study design. Second, PAF is based on multiple assumptions. One of these assumptions is that PAF assumes that risk factors precede and be causally related to the outcome. This assumption requires a longitudinal study design–a prospective cohort study. Ignoring such assumptions can lead to inaccurate estimations and hence incorrect interpretation of PAF estimates. Lastly, length of followup is another critical factor in accurately valuing PAF. In this systematic review, the follow up period ranged from 5 to 20 years overall and from 7 to 12 years in the three prospective cohort studies. Importantly, short follow up times tend to overestimate PAF and longer followup times generally underestimate PAF [21].

#### Measurement of exposure (domain-specific PAF)

Another reason that could explain the observed degree of variation in PAF is the use of different definitions for the physical inactivity. Physical inactivity occurs when total activity fails to meet the recommended guidelines of ≥ 150 minutes of moderate-intensity or ≥ 75 minutes of vigorous-intensity aerobic physical activity per week in bouts of at least 10 minutes duration accumulated across occupational, transport-related, domestic or leisure-time activity domains [5]. Leisure–time activity consists of exercise, sport [14,25,26]. Specifically, exercise and sports are unique subsets of the leisure-time activity domain; they are not interchangeable [27]. Therefore, acknowledging distinction between is essential in our interpretation of results [27]. Two studies reporting three prospective cohorts scored high in our quality assessment. Despite this, the PAF estimates varied widely from 13% (3, 22) to 29% (17, 41) for occasional exerciser (≤30 min/day) [13] and 3% (-11, 16) to 7% (-9, 20) for non sport participants. This could partially be explained by the use different subsets definition of leisure-time domain. In the four country-specific reviews, only two studies [41,42] used similar definitions for the physical inactivity of the leisure-time activity domain. In the two global review studies, the PAFs ranged from 5.2% (2.2, 8.2) to 10.9% (4.8, 16.6). These studies [14,26] also have different definitions for physical inactivity. For example, Bull [26] estimated PAF for total physical inactivity while Lee [14] estimated PAF based primarily on the leisure-time domain.

Another factor that could explain variation in PAF is that physical activity was self reported in all studies except one [37]. A higher PAF of 39% (35.9, 41.7) was based on Canadian data [15]. One explanation for the higher PAF observed may be due in part to how physical inactivity is assessed. For example, using an objective measure such as accelerometry is more likely to capture total physical activity compared than a subjective measure (i.e., self report). Self reporting of physical inactivity is prone to measurement error (i.e., often underestimation of physical inactivity) and consequently biased PAF (i.e., often overestimation) estimates. In a systematic review, Prince [35] reported low-to-moderate correlations between self-report and direct measures of physical inactivity that ranged from -0.71 to 0.96. A clear trend for the mean differences was not present. However, self-report measures were 44% (range: -78% to 500%) higher than those measured directly by accelerometers. This suggests there is a trend of self-report measures over reporting physical activity leading to an under-estimation of both physical inactivity and subsequent PAF estimates.

#### Measurement of outcome

A third reason that could explain PAF estimate variation is the use of different definitions for T2DM [30,45]. Current diagnostic criteria are fasting plasma ≥ 7.0 mmol/l (126 mg/dl) or 2-h plasma glucose ≥ 11.1 mmol/l (200 mg/dl) [29,30]. Among the studies we reviewed, there were some differences in methods of diagnosis of T2DM. None of the studies included in this review was based solely on plasma glucose.

Self- reported T2DM is also subject to measurement bias. For instance, the accuracy of self-reported T2DM is good (kappa = 0.78) and of moderate sensitivity (73%) [46,47]. However, T2DM can remain asymptomatic for at least 4 to 7 years before a clinical diagnosis is made.[48]. As a result, T2DM may be undiagnosed in up to 50% of cases [49,50]. This underestimation of the incidence of T2DM leads to an underestimate of RR and PAF. Therefore, objective measurement of T2DM is desirable for accurate PAF estimates.

### Statistical analysis

There are two published modeling techniques PAF: the full adjustment method and the partial adjustment method. Below we discuss the pros and cons of these methods in the context of estimating the PAF of T2DM attributable to physical inactivity.

#### Full adjustment method (modeling techniques)

In the two prospective studies PAF different modeling techniques were used. Laaksonen used a piecewise constant hazard model while Steinbrecher used Cox proportional hazard model [21,24]. To reduce bias in PAF estimates and account for death, Laaksonen [21] suggests using piecewise over Cox model when the outcome of interest is disease.

#### Partial adjustment method (crude formula)

In the four country-specific review studies, the PAF was calculated from published data of adjusted relative risk (RRadj) using previously published cohort studies and the prevalence of physical inactivity (prevalence of exposure, Pe) was estimated from previously published cross sectional surveys. In the presence of confounding, a popular method of calculating PAF is to use published adjusted RR and estimated prevalence in the crude formula 1 [31]:

$$\mathrm{PAF}=\frac{P_{e}\left(\mathrm{RRadj}‒1\right)}{\left[\mathrm{Pe}\;\left(\mathrm{RRadj}‒1\right)\right]+1}\times \;100$$

This method is called partial adjustment. Partial adjustment is a common method when data on all known confounders are not available or not measured. However, formula 1 should only be used in the absence of confounding, because it assumes no confounding of the exposure-outcome association [25]. Four of the country specific review studies in this review used formula 1. In the presence of confounding another variant formula is recommended, formula 2 [31]:

$$\mathrm{PAF}=\frac{P_{e}\left(\mathrm{RRadj}‒1\right)}{\mathrm{RRadj}}\times \;100$$

Only one global review study [14] used formula 2. Severe confounding bias may occur with partial adjustment method, especially formula 1, because the fraction of the outcome that is attributable to the confounding variables is not adequately adjusted [36]. For example, one study demonstrated that partial adjustment for confounding by age and sex yielded a 17% overestimation in PAF [36]. Hence, the full adjustment method that adjusts for all known confounders is a better choice for estimating PAF.

#### Adjustment for confounders

In this review, over-adjustment or under-adjustment (most likely) of known confounders varied explaining some of the variation in PAF estimates [51]. For instance, adjusting for intermediate variables as confounders can lead to over-estimated or null-biased PAF [52]. Therefore, adjustment should be limited to known evidence based confounders.

### Subgroup analysis (sex specific PAF)

PAF integrates and is directly related to relative risk (RR) and the prevalence of physical inactivity (Pe) in the population [53]. Thus, for a given RR, different prevalence estimates for physical inactivity yield different PAF estimates and vice versa in a non linear fashion [53]. In this review, one high quality prospective study reported widely variable sex specific PAFs for non sport participants [40]: 29% (95% CI: 17, 41) for women and 13% (95% CI: 3, 22) for men. In women, both the RRadj 1.43 (95% CI: 1.21, 168) and Pe 66.3% were higher than men: RRadj 1.21 (1.1, 1.35) and Pe 55.2%, respectively. This could explain sex difference observed in PAF estimates. For example, Flegal [36] showed that a small difference of 3% in age subgroup between the source population and the target population lead to a 42% overestimation in PAF. In addition PAF is sensitive to minor changes in RR. A difference of 0.20 in RR almost doubled the PAF estimate. This highlights the important of accurately quantifying the RR and Pe prior to estimating PAF.

### Limitations and strengths

This systematic review did not include a meta-analysis because pooling was not appropriate due to the heterogeneity of studies at conceptual, operational, design and statistical levels. Study heterogeneity was due in part to the inclusion criteria for this systematic review. Specifically, we included studies that estimated PAF or PAR using modeling on raw data from a prospective cohort design or (ii) that used published adjusted relative risk. Further, data from each study on physical inactivity were collected from different populations using different sampling and estimation methods. These differences contribute to the wide variation in PAF T2DM attributable to physical inactivity. This is the first systematic review that has ascertained the PAF T2DM attributable to physical inactivity. We believe the results of this systematic review provide an essential platform for understanding methodological and statistical reasons that underpin current and widely varying PAF estimates. Further, this study provides an initial step toward developing criteria to report and evaluate PAFs in the future.

## Conclusions

The best quality data from this systematic review indicate the PAF of T2DM attributable to physical inactivity should be considered and interpreted by domain and/or subset of physical inactivity. In the USA, PAFs for sport ranged from 13% (95% CI: 3, 22) to 29% (95% CI: 17, 41): 13% (95% CI: 3, 22) in men and 29% (95% CI: 17, 41) in women. In Finland, the PAFs for exercise ranged from 3% (95% CI: -11, 16) to 7% (95% CI: -9, 20). The best study design for estimating PAF is the prospective cohort. To obtain the most accurate estimate of PAF the following need to be implemented: objective measurement for exposure (physical inactivity), objective measurement of outcome (T2DM), full adjustment method that adjusted for all known confounder and a piecewise model.

PAF is a valuable statistic in ascertaining burden of a disease due to a specific risk factor from a public health perspective only when it is accurately calculated using an appropriate study design (i.e., a prospective cohort study). Future studies estimating PAF could reduce the wide variability we currently observe in PAF data by using valid and reliable methods to measures physical inactivity and by using consistent ‘best practice’ methodology for reporting PAF [21,54]. Such improvements in study design methodology and consistent cutting edge methodology will facilitate appropriate and well-informed public health decision making choices.

## Competing interests

The authors declare that they have no competing interests.

## Authors’ contributions

HAT and JCD searched for relevant literature and wrote the manuscript. Together with KMK, they conceived the study idea. KMK and DCM helped with drafting and revisions. KMK has given the final approval of the version to be published. All authors read and approved the final manuscript.

## Pre-publication history

The pre-publication history for this paper can be accessed here:

http://www.biomedcentral.com/1471-2458/14/469/prepub

## Supplementary Material

Additional file 1: Table S1 Characteristics of studies and outcome measure. Click here for file

Additional file 2: Table S2 Summary estimate of prevalence of exposure (Pe), adjusted relative risk (RRadj), population attributable fraction (PAF) and calculation methods of PAF for physical inactivity domains. Click here for file

Additional file 3: Table S3 Quality assessment* of the eight studies. Click here for file
