# Supplementary material for: Population attributable fraction of type 2 diabetes due to physical inactivity in adults: a systematic review
Source: BMC Public Health. 2014 May 18;14:469. doi: 10.1186/1471-2458-14-469 (PMC4083369; doi:10.1186/1471-2458-14-469)
Supplement: Additional file 1: Table S1 — Characteristics of studies and outcome measure. [file 1471-2458-14-469-S1.docx]

| **Additional file 1: Table S1:** Characteristics of studies and outcome measure | | | | |
| --- | --- | --- | --- | --- |
| Author,  Year,  Country,  Study design) | Study sample,  Total number of participants,  Age,  Data collection year,  Number of follow up years | Exposure  Physical inactivity:  Definition,  Measurement,  Categorization | Outcome  Diabetes Type 2:  Definition,  Ascertainment,  Activity level classification for RRadj | Confounders |
| **Bull** [1]**,**  2000,  Global report,  WHO,  Review on published data | **Pe source:**  Meta-analysis of 21 data sets covering 3 countries across 13 sub-regions  137775 participants  Aged 18+ years  1996-2000  **RRadj source:**  Meta-analysis: 23 prospective cohort studies  315757 participants  Aged: 20+  2004  Follow up time: 5-17 years | **Definition:** adjusted for WHO, total physical inactivity: < 150 min of moderate or 60 min of vigorous intensity/w accumulated across work, home, transport or discretionary domains,  **Measurement:** self reported total physical inactivity adjusted for measurement error,  **Categorization:** WHO 3 categories (C). C1: inactive (C1): not meeting C2 or C3 criteria, insufficiently active (C2): < 150 min of moderate or 60 min of vigorous intensity physical activity/w. sufficiently active (C3): > 150 min of moderate or 60 min of vigorous intensity physical activity/w | **Definition:** hyperosmolarity without nonketotic hyperglycemic-hyperosmolar coma,  **Ascertainment:** inconsistent, oral glucose tolerance test (OGTT), fasting plasma glucose test, self reported, medical record. Adjustment effort was made to address measurement error and heterogeneity,  **Activity level classification for RRajd:** inconsistent but adjustment effort was made to address definition, measurement error and heterogeneity that is consistent with WHO definition and classification | Adjusted for variety of confounders or intermediary factors: age, BMI or waist/hip ratio, blood pressure, cholesterol and family history |
| **Janssen** [2]**,** 2012,  Canada,  Applied physiology, nutrition, and metabolism, Country specific review on published data | **Pe source:**  Canadian Health Measures Survey (CHMS)  National representative 2832 participants  Aged: 20-79 years  2007-2009  **RRadj source:**  Meta-analysis: 20 prospective cohort studies  624952 participants  Aged: 30+  2010  Follow up time: 3-16.8 years | **Definition:** < 150 min/w of moderate-vigorous physical activity accumulating in bouts of at least 10 min or 10,000 steps/d, **Measurement:** accelerometer (7 d)  **Categorization:** sedentary (< 2 METs), light (2-2.9 METs), moderate (3-5.9 METs, vigorous (> 6 METs) | **Definition:** not provided  **Ascertainment:** inconsistent, self reported, clinical & medical records, registries  **Activity level classification for RRadj:** daily life, walking, leisure-time (sports, exercise), occupational (commuting, transportation), total (MET/w or Kcal/w), sedentary (sitting time), fitness (max treadmill test, submax cycle ergometer) | Variable degree of adjustment for confounders: age, smoking, alcohol menopausal status, postmenopausal hormone use, parental history, history of hypercholesterolemia or hypertension, BMI, ethnicity, gender, income, educational level, skin fold, lipids, hyperuricemia |
| **Joubert** [3]**,** 2007,  South Africa, South African medical journal, Country specific review on published data | **Pe source:**  World Health Survey (WHS)  Aged: 18+  2003  **RRadj source:**  Meta-analysis: 23 prospective cohort studies  315757 participants  Aged: 20+  2004  Follow up time: 5-17 years | **Definition:** < 150 min of moderate or 60 min of vigorous intensity/w accumulated across work, home, transport or discretionary domains, **Measurement:** self reported (IPAQ: International Physical Activity Questionnaire),  **Categorization:** WHO 3 categories (C). C1: inactive; not meeting C2 or C3. C 2: insufficiently active; < 150 min of moderate or 60 min of vigorous intensity/w. C3: sufficiently active; > 150 min of moderate or 60 min of vigorous intensity/w | **Definition:** hyperosmolarity without nonketotic hyperglycemic-hyperosmolar coma  **Ascertainment:** inconsistent, oral glucose tolerance test (OGTT), fasting plasma glucose, self reported, medical record  **Activity level classification for RRadj:** inconsistent but adjustment effort was made to address definition, measurement error and heterogeneity that is consistent with WHO definition and classification | Adjusted for variety of important confounders or intermediary factors including: age, BMI, blood pressure, cholesterol and family history |
| **Katzmarzyk** [4], 2004**,** Canada, Canadian journal of applied physiology, Country specific review on published date, Update | **Pe source:**  Canadian Community Health Survey (CCHS)  National representative: 136 health regions, 133300  Aged 12+ years,  2000-2001  **RRadj source:**  Meta-analysis: 11 prospective cohort studies  207550 participants  Aged: 18+  2004  Follow up time: 4-20 years | **Definition:** < 12.6 kJ/kg/d or < 3 kcal/kg/d of physical activity ~ 1 mile (1.6) walk/d  **Measurement:** computer assisted interviewing  **Categorization:** leisure-time; active: >3 kcal/kg/d, moderately active: 1.5-3, inactive < 1.5 | **Definition:** not provided  **Ascertainment:** inconsistent, self reported, clinical & medical records, registries  **Activity level classification for RRajd:** daily life, walking, leisure-time (sports, exercise), total (MET/w or Kcal/w) | Variable degree of adjustment for confounders: age, smoking, alcohol menopausal status, postmenopausal hormone use, parental history, history of hypercholesterolemia or hypertension, BMI, ethnicity, gender, income, educational level, skin fold, lipids, hyperuricemia |
| **Katzmarzyk** [5]**,** (2000), Canada, Canadian Medical Association journal, Country specific review on published data | **Pe source:**  Physical Activity Monitor Survey  Representative 1875 Canadians  Aged 18+ years  1996-1997  **RRadj source:**  Meta-analysis: 4 prospective cohort studies  138910 participants  Aged: 18+  2000  Follow up time: 4-20 years | **Definition:** < 12.6 kJ/kg/d or < 3 kcal/kg/d of physical activity ~ 1 mile (1.6) walk/d  **Measurement:** computer assisted interviewing  **Categorization:** leisure-time; active: >3 kcal/kg/d, moderately active: 1.5-3, somewhat active: 0.5-1.5, sedentary < 0.5 | **Definition:** not provided  **Ascertainment:** inconsistent self reported, clinical & medical records, registries  **Activity level classification for RRadj:** daily life, walking, leisure-time (sports, exercise) with intensity, duration, frequency | Variable degree of adjustment for confounders: age, smoking, alcohol menopausal status, postmenopausal hormone use, parental history, history of hypercholesterolemia or hypertension, BMI, ethnicity, gender, income, educational level, skin fold, lipids, hyperuricemia |
| **Laaksonen** [6]**,** (2010), Finland, European journal of epidemiology, Cohort | Health 2000 survey  8028 individuals (80 geographical areas, 84% participation)  Aged: 40-79 years  2000-2001  Follow up time: 7 years | **Definition:** exercise (≥ 30 min/d)  **Measurement:** self reported  **Categorization:** occasional exercise (≤ 30 min/d), regular exercise (≥ 30 min/d) | **Definition:** WHO diagnostic criteria (1985): fasting (≥7.8 mmo/l or 140 mg/dl), 2-hr (≥11.1 mmol/l or 200 mg/dl)  **Ascertainment:** self reported, confirmed by medication, reimbursement  **Cumulative incidence:** 65/1646= 3.9 per 100 person | Sex, age, BMI (<25 kg/m^2^), alcohol (men: 1-99 g/w, women: 1-199 g/w), smoking (not smoking), serum-vitamin D (>44 nmol/l) |
| **Laaksonen** [6]**,** (2010), Finland, European journal of epidemiology, Cohort | Mini-Finland Health (MFH): 8000 individuals (40 geographical areas, 90% participation)  40-79 years  1978-1978 | **Definition:** exercise (≥ 30 min/d)  **Measurement:** self reported  **Categorization:** occasional exercise (≤ 30 min/d), regular exercise (≥ 30 min/d) | **Definition:** WHO diagnostic criteria (1985): fasting (≥7.8 mmo/l or 140 mg/dl), 2-hr (≥11.1 mmol/l or 200 mg/dl), confirmed by medication reimbursement, **Ascertainment:** self reported  **Cumulative incidence:** 26/970= 2.6 per 100 person | Sex, age, smoking (not smoking), alcohol (men: 1-99 g/w, women: 1-199 g/w), BMI (<25 kg/m^2^), serum-vitamin D (>39 nmol/l) |
| **Lee** [7]**,** (2012)**, Gl**obal report, Lancet, review on published data | **Pe source:**  Global risk factors surveillance  Aged: 15+ standardized by country  2008  **RRadj source:**  Meta-analysis: 10 prospective cohort studies  301,221 participants  Aged: 35+  2006  Follow up time: 4-17 years | **Definition:** < 5 times 30 min of moderate activity/w, or < 3 times 20 min of vigorous activity/w, or equivalent (WHO 2010 guidelines), mostly leisure-time activity, **Measurement:** self reported, standardized to WHO questionnaire  **Categorization:** low, moderate and high intensity physical activity | **Definition:** fasting plasma glucose ≥ 7.0 mmol/L (126 mg/dl) or medication for raised blood glucose,  **Ascertainment:** inconsistent self reported, OGTT, primary care records, national registry  **Activity level classification for RRadj:** inconsistent | Variable degree of adjustment level for age, BMI, duration of vigorous activities, smoking, menopausal status, hormone therapy, family history, alcohol, hypertension, hypercholesterolemia, waist to hip ratio, education, smoking, diet, weekday physical activity, social class, pre-existing coronary heart disease, study year, systolic hypertension, other physical activity |
| **Steinbrecher** [8]**,** (2011), USA, Journal of physical activity & health, Cohort | Multiethnic cohort (Hawaii & California),  74970 (M: 36075, F: 38895, response rate 28-51%)  Aged 45-75 years  1993-1996  Follow up time (mean): 12.1 years | **Definition:** mean h/w of strenuous sports (jogging, tennis, aerobics)  **Measurement:** self reported  **Categorization:** never, ½ -1, 2-3, ≥ 4 h/w | **Definition:** not provided  **Ascertainment:** self reported, medication confirmed by health plan  **Cumulative incidence:** 2768+2938/74970= 7.6 per 100 person) | Age, gender, ethnicity, education, smoking, BMI, diet, alcohol |
| BMI: body mass index, d: day, h: hour, Kcal: kilo calorieMET: metabolic equivalent of task, min: minute, w: week, WHO: world health organization, y: year,  ^a^Substitution method was used to constructed 95% CI for PAF  ^b^Estimated from WHO report for Africa (AFR-E): age group 15-69 years | | | | |

References

1. Bull FC, Armstrong TP, Dixon T, Ham S, Neiman A, Pratt M: **Physical inactivity.** In *Comparative quantification of health risks. Volume 1.* Edited by Ezzati M, Lopez A, Rodgers A, Murray C.  World Health Organization; 2004:729-882.

2. Janssen I: **Health care costs of physical inactivity in Canadian adults.** *Applied Physiology, Nutrition, and Metabolism* 2012, **37**(4):803-806.

3. Joubert J, Norman R, Lambert EV, Groenewald P, Schneider M, Bull F, Debbie B: **Estimating the burden of disease attributable to physical inactivity in South Africa in 2000.** *South African Medical Journal* 2007, **97**:725+.

4. Katzmarzyk PT, Janssen I: **The Economic Costs Associated With Physical Inactivity and Obesity in Canada: An Update.** *Can J Appl Physiol* 2004, **29**(1):90-115.

5. Katzmarzyk PT, Gledhill N, Shephard RJ: **The economic burden of physical inactivity in Canada.** *Canadian Medical Association Journal* 2000, **163**(11):1435-1440.

6. Laaksonen MA, Knekt P, Rissanen H, Härkänen T, Virtala E, Marniemi J, Aromaa A, Heliövaara M, Reunanen A: **The relative importance of modifiable potential risk factors of type 2 diabetes: a meta-analysis of two cohorts.** *Eur J Epidemiol* 2010, **25**(2):115-124.

7. Lee I, Shiroma EJ, Lobelo F, Puska P, Blair SN, Katzmarzyk PT: **Effect of physical inactivity on major non-communicable diseases worldwide: an analysis of burden of disease and life expectancy.** *The Lancet* 2012, **380**(9838):219-229.

8. Steinbrecher A, Morimoto Y, Heak S, Ollberding NJ, Geller KS, Grandinetti A, Kolonel LN, Maskarinec G: **The preventable proportion of type 2 diabetes by ethnicity: the multiethnic cohort.** *Ann Epidemiol* 2011, **21**(7):526-535.
