# Supplementary material for: Population attributable fraction of type 2 diabetes due to physical inactivity in adults: a systematic review
Source: BMC Public Health. 2014 May 18;14:469. doi: 10.1186/1471-2458-14-469 (PMC4083369; doi:10.1186/1471-2458-14-469)
Supplement: Additional file 2: Table S2 — Summary estimate of prevalence of exposure (Pe), adjusted relative risk (RRadj), population attributable fraction (PAF) and calculation methods of PAF for physical inactivity domains. [file 1471-2458-14-469-S2.docx]

| **Additional file 2: Table S2:** Summary estimate of prevalence of exposure (Pe), adjusted relative risk (RRadj), population attributable fraction (PAF) and calculation methods of PAF for physical inactivity domains | | | | |
| --- | --- | --- | --- | --- |
| Publication  (Author,  Year,  Country,  Journal,  Design) | Prevalence of exposure (Pe%) | Adjusted RR  (RRadj)  95% CI | Domain specific PAF%  (95%CI) | PAF  Calculation method |
| **Bull** [1]**,** 2000,  Global report,  WHO,  Global review on published data | Global: 40.6  USA: 45  Canada: 23  Finland: 63  South Africa: 51 | 1.24  (1.1, 1.39) | Domain: total (all domains)  Global: 14  Unable to construct 95% CI^a^  USA: 9.7 (4.3, 14.9)  Canada: 5.2 (2.2, 8.2)^a^  Finland: 13 (5.9, 19.6)^a^  South Africa: 10.9 (4.8, 16.6)^a^ | Published RRadj & prevalence of inactivity (Pe) plugged into PAF crude formula:  Pe(RRadj-1) x 100  [Pe(RRadj-1)] +1 |
| **Janssen** [2]**,** 2012,  Canada,  Applied physiology, nutrition, and metabolism,  Country specific review on published data | Men: 82.9  Women: 86.3 | 1.74  (1.65, 1.83) | Domain: total (all domains)  Men:  38  (35, 40.7)^a^  Women:  39  (35.9, 41.7)^a^ | Published RRadj & prevalence of inactivity (Pe) plugged into PAF crude formula:  Pe(RRadj-1) x100  [Pe(RRadj-1)] + 1 |
| **Joubert** [3]**,** 2007,  South Africa,  South African medical journal,  Country specific review on published data | Men: 19.8  Women: 26.8 | 1.24  (1.1, 1.39) | Domain: total (all domains)  Men:  20.4  (15.7, 26)^a^  Women:  20.1  (17.8, 30.1)^a^ | Published RRadj & prevalence of inactivity (Pe) plugged into PAF crude formula:  Pe(RRadj-1) x 100  [Pe(RRadj-1)] +1 |
| **Katzmarzyk** [4], 2004**,** Canada,  Canadian journal of applied physiology,  Country specific review on published date, Update | 53.5 | 1.5  (1.37, 1.63) | Domain: leisure-time  21.1  (16.5, 25.2)^a^ | Published RRadj & prevalence of inactivity (Pe) plugged into PAF crude formula:  Pe(RRadj-1) x 100  [Pe(RRadj-1) ]+ 1 |
| **Katzmarzyk** [5]**,** (2000), Canada,  Canadian Medical Association journal,  Country specific review on published data | 62 | 1.4  (1.2, 1.6) | Domain: leisure-time  19.9  (11, 27.1)^a^ | Published RRadj & prevalence of inactivity (Pe) plugged into PAF crude formula:  Pe(RRadj-1) x 100  [Pe(RRadj-1) ]+ 1 |
| **Laaksonen** [6]**,** (2010), Finland,  European journal of epidemiology,  Cohort | 24.1 | 1.35  (.97, 1.6) | Domain: leisure-time; exercise subset  7  (-9, 20) | Piecewise constant hazard ratio, logarithmic transformation, delta method for 95% CI, censored for T2DM, death and lost follow up |
| **Laaksonen** [6]**,** (2010),  Finland,  European journal of epidemiology,  Cohort | 36.5 | 1.28  (.99, 1.48) | Domain: leisure-time; exercise subset  3  (-11, 16) | Piecewise constant hazard ratio, logarithmic transformation, delta method for 95% CI, censored for T2DM, death and lost follow up |
| **Lee** [7]**,** (2012)**,**  Global report,  Lancet,  Global review on published data | Global: 35.2  USA: 43.2  Canada: 35.7  Finland: 40.8  South Africa: 51.1 | 1.2  (1.1, 1.33) | Domain: leisure-time  Global: 7.2 (3.9, 9.6)  USA: 8.3 (4.2, 12.9)  Canada: 7 (0.8, 14.4)  Finland: 7.8 (1.3, 15.6)  South Africa: 10.7 (5.4, 16.8) | Published RRadj & prevalence of inactivity (Pe) plugged into PAF crude formula:  Pe(RRadj-1) x 100  RRadj |
| **Steinbrecher** [8]**,**  (2011),  USA,  Journal of physical activity & health,  Cohort | Men  55.2  Women  66.3 | Men:  1.21  (1.1, 1.35)  Women:  1.43  (1,21, 1.68) | Domain: leisure-time; sport subset  Men:  13  (3, 22)  Women:  29  (17, 41) | Cox proportional hazard model |
| ^a^ The authors used a substitution method described in the methods section to calculate the 95%CI for the domain specific PAF% | | | | |

References

1. Bull FC, Armstrong TP, Dixon T, Ham S, Neiman A, Pratt M: **Physical inactivity.** In *Comparative quantification of health risks. Volume 1.* Edited by Ezzati M, Lopez A, Rodgers A, Murray C.  World Health Organization; 2004:729-882.

2. Janssen I: **Health care costs of physical inactivity in Canadian adults.** *Applied Physiology, Nutrition, and Metabolism* 2012, **37**(4):803-806.

3. Joubert J, Norman R, Lambert EV, Groenewald P, Schneider M, Bull F, Debbie B: **Estimating the burden of disease attributable to physical inactivity in South Africa in 2000.** *South African Medical Journal* 2007, **97**:725+.

4. Katzmarzyk PT, Janssen I: **The Economic Costs Associated With Physical Inactivity and Obesity in Canada: An Update.** *Can J Appl Physiol* 2004, **29**(1):90-115.

5. Katzmarzyk PT, Gledhill N, Shephard RJ: **The economic burden of physical inactivity in Canada.** *Canadian Medical Association Journal* 2000, **163**(11):1435-1440.

6. Laaksonen MA, Knekt P, Rissanen H, Härkänen T, Virtala E, Marniemi J, Aromaa A, Heliövaara M, Reunanen A: **The relative importance of modifiable potential risk factors of type 2 diabetes: a meta-analysis of two cohorts.** *Eur J Epidemiol* 2010, **25**(2):115-124.

7. Lee I, Shiroma EJ, Lobelo F, Puska P, Blair SN, Katzmarzyk PT: **Effect of physical inactivity on major non-communicable diseases worldwide: an analysis of burden of disease and life expectancy.** *The Lancet* 2012, **380**(9838):219-229.

8. Steinbrecher A, Morimoto Y, Heak S, Ollberding NJ, Geller KS, Grandinetti A, Kolonel LN, Maskarinec G: **The preventable proportion of type 2 diabetes by ethnicity: the multiethnic cohort.** *Ann Epidemiol* 2011, **21**(7):526-535.
