# Supplementary material for: Population attributable fraction of type 2 diabetes due to physical inactivity in adults: a systematic review
Source: BMC Public Health. 2014 May 18;14:469. doi: 10.1186/1471-2458-14-469 (PMC4083369; doi:10.1186/1471-2458-14-469)
Supplement: Additional file 3: Table S3 — Quality assessment* of the eight studies. [file 1471-2458-14-469-S3.docx]

| **Additional file 3: Table S3:** Quality assessment* of the eight studies | | | | | | | |
| --- | --- | --- | --- | --- | --- | --- | --- |
| **Authors, Year** | 1 | 2 | 3 | 4 | 5 | 6 | 7 |
| Bull [1] , 2000 | + | - | + | - | - | + | - |
| Janssen[2], 2012 | + | + | - | - | - | + | - |
| Joubert [3], 2007 | + | - | + | - | - | - | - |
| Katzmarzyk [4], 2004 | + | - | - | - | - | + | - |
| Katzmarzyk [5], 2000 | + | - | - | - | - | + | - |
| Laaksonen [6], 2010^a^ | + | - | + | + | + | + | + |
| Laaksonen [6], (2010^b^ | + | - | + | + | + | + | + |
| Lee [7], 2012 | + | - | + | - | - | + | - |
| Steinbrecher [8], 2011 | + | - | - | + | + | + | + |

+ Study met the criterion

- All or part of the criterion were not met

^a^ Mini Finland Health (MFH) survey

^b^ Health 2000 survey

*The following 7 criteria of the checklist comprised our quality assessment checklist:

1. Was clear definition provided for the exposure (physical inactivity)?
2. Was the exposure (physical inactivity) measured objectively?
3. Was clear definition provided for the outcome (T2DM)?
4. Was the outcome ascertained by objective measures or if self reported confirmed by other measures (T2DM)?
5. Was the analysis based on raw data from prospective cohort study?
6. Was the follow up time provided?
7. Was population attributable fraction (PAF) or proportion (PAR) fully adjusted?

References

1. Bull FC, Armstrong TP, Dixon T, Ham S, Neiman A, Pratt M: **Physical inactivity.** In *Comparative quantification of health risks. Volume 1.* Edited by Ezzati M, Lopez A, Rodgers A, Murray C.  World Health Organization; 2004:729-882.

2. Janssen I: **Health care costs of physical inactivity in Canadian adults.** *Applied Physiology, Nutrition, and Metabolism* 2012, **37**(4):803-806.

3. Joubert J, Norman R, Lambert EV, Groenewald P, Schneider M, Bull F, Debbie B: **Estimating the burden of disease attributable to physical inactivity in South Africa in 2000.** *South African Medical Journal* 2007, **97**:725+.

4. Katzmarzyk PT, Janssen I: **The Economic Costs Associated With Physical Inactivity and Obesity in Canada: An Update.** *Can J Appl Physiol* 2004, **29**(1):90-115.

5. Katzmarzyk PT, Gledhill N, Shephard RJ: **The economic burden of physical inactivity in Canada.** *Canadian Medical Association Journal* 2000, **163**(11):1435-1440.

6. Laaksonen MA, Knekt P, Rissanen H, Härkänen T, Virtala E, Marniemi J, Aromaa A, Heliövaara M, Reunanen A: **The relative importance of modifiable potential risk factors of type 2 diabetes: a meta-analysis of two cohorts.** *Eur J Epidemiol* 2010, **25**(2):115-124.

7. Lee I, Shiroma EJ, Lobelo F, Puska P, Blair SN, Katzmarzyk PT: **Effect of physical inactivity on major non-communicable diseases worldwide: an analysis of burden of disease and life expectancy.** *The Lancet* 2012, **380**(9838):219-229.

8. Steinbrecher A, Morimoto Y, Heak S, Ollberding NJ, Geller KS, Grandinetti A, Kolonel LN, Maskarinec G: **The preventable proportion of type 2 diabetes by ethnicity: the multiethnic cohort.** *Ann Epidemiol* 2011, **21**(7):526-535.
